# Dose individualisation of antibiotics in critically ill patients with inflammation: A narrative review

**DOI:** 10.1002/bcp.70185

**Published:** 2025-08-14

**Authors:** Sarah Dräger, Iris K. Minichmayr, Narges Alipanah‐Lechner, Erin F. Barreto, Lieuwe D. J. Bos, Lucas M. Fleuren, Nicole G. M. Hunfeld, Sumith K. Mathew, Sophie L. Stocker, João Paulo Telles, Antoni Torres, Birgit C. P. Koch, Henrik Endeman

**Affiliations:** ^1^ Department of Hospital Pharmacy Erasmus University Medical Center Rotterdam The Netherlands; ^2^ Rotterdam Clinical Pharmacometrics Group Rotterdam The Netherlands; ^3^ Department of Internal Medicine University Hospital Basel Basel Switzerland; ^4^ Department of Clinical Pharmacology Medical University of Vienna Vienna Austria; ^5^ Division of Pulmonary, Critical Care, Allergy, and Sleep Medicine, Department of Medicine University of California San Francisco CA USA; ^6^ Department of Pharmacy Mayo Clinic Rochester Minnesota USA; ^7^ Department of Intensive Care Amsterdam University Medical Centers, Location 'AMC' Amsterdam The Netherlands; ^8^ Department of Respiratory Medicine Amsterdam University Medical Centers, Location 'AMC' Amsterdam The Netherlands; ^9^ Department of Intensive Care Medicine Erasmus University Medical Center Rotterdam The Netherlands; ^10^ Department of Pharmacology and Clinical Pharmacology Christian Medical College Vellore India; ^11^ School of Pharmacy Faculty of Medicine and Health, The University of Sydney Sydney NSW Australia; ^12^ Sydney Institute for Infectious Diseases The University of Sydney Sydney NSW Australia; ^13^ Department of Clinical Pharmacology and Toxicology St. Vincent's Hospital Sydney NSW Australia; ^14^ Department of Infectious Diseases AC Camargo Cancer Center São Paulo‐SP Brazil; ^15^ CIBER of Respiratory Diseases (CIBERES), Institute of Health Carlos III Madrid Spain; ^16^ Department of Pneumology Hospital Clinic of Barcelona‐August Pi i Sunyer Biomedical Research Institute (IDIBAPS), University of Barcelona Barcelona Spain

**Keywords:** antibiotics, critically ill, dosing, inflammation, pharmacokinetics and pharmacodynamics, therapeutic drug monitoring

## Abstract

Due to extensive pathophysiological changes in critically ill patients, standard dosing of antibiotics may lead to inadequate drug exposure. This is concerning, as insufficient plasma drug concentrations may lead to treatment failure, whereas excessive drug exposure may increase the risk of toxic adverse events. The role of inflammation as a factor influencing the pharmacokinetics (PK) and pharmacodynamics (PD) of antibiotics remains largely unknown. PK/PD target attainment of antibiotics can be improved through therapeutic drug monitoring, i.e., measurement of drug concentrations in the blood with subsequent dosage adjustment to reach a certain target. Besides, population PK models may be used to predict drug exposure and tailor dosing in an individual patient (model‐informed precision dosing). Inflammatory biomarkers have been proposed to measure inflammation levels and guide antibiotic treatment. However, their potential to guide antibiotic dosing is unclear. This narrative review describes associations between inflammation and PK/PD of antibiotics in critically ill patients, and the role of biomarkers, therapeutic drug monitoring and model‐informed precision dosing in improving antibiotic dosing. A focus of future research should be on the interplay between inflammation and PK/PD of antibiotics by including inflammatory biomarkers in PK/PD models and using big data to predict antibiotic exposure in critically ill patients.

## 1 INTRODUCTION

**FIGURE 1:**
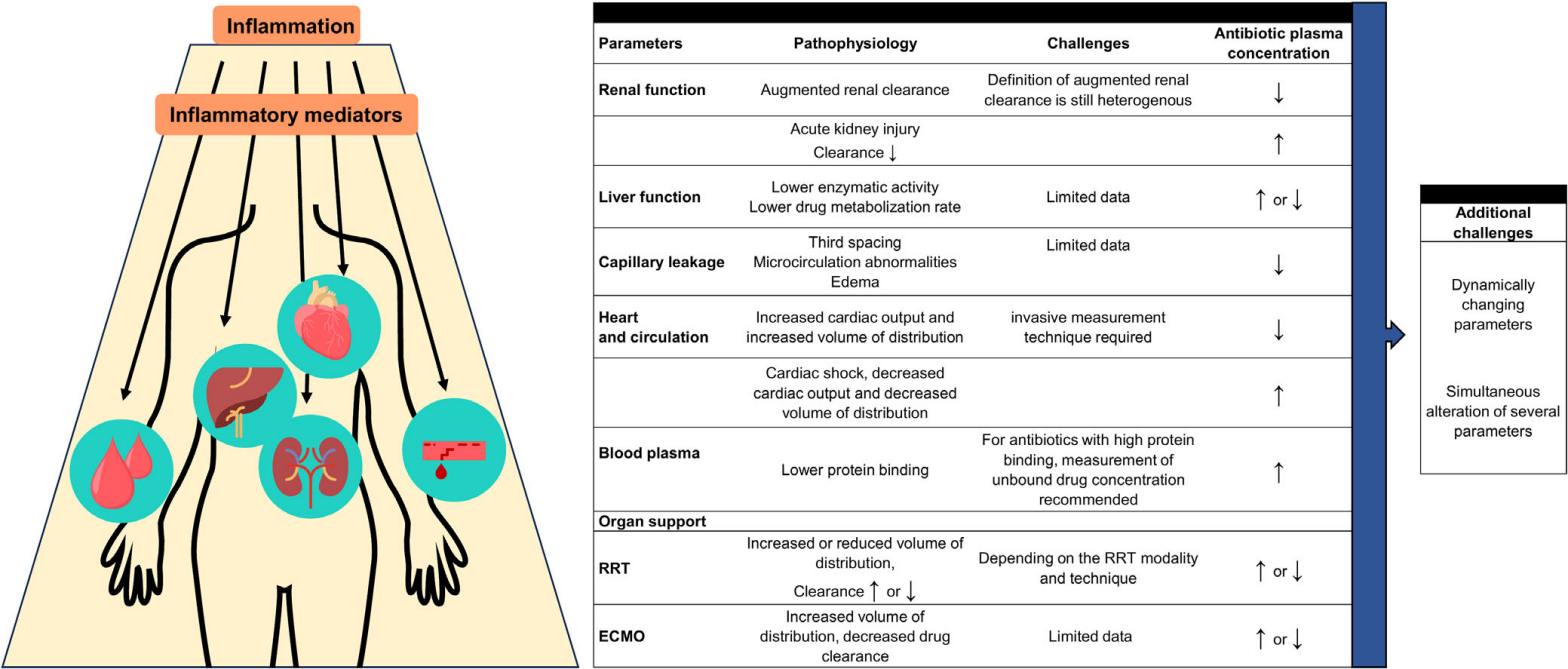
Pathophysiological changes in critically ill patients with inflammation and their potential impact on antibiotic plasma concentrations. ECMO, extracorporeal membrane oxygenation; eGFR, estimated glomerular filtration rate; RRT, renal replacement therapy.

Timely and appropriate antibiotic treatment is crucial for critically ill patients with sepsis and septic shock.^1^ However, this patient population is highly heterogeneous, exhibiting extensive pathophysiological changes such as organ dysfunction, systemic inflammation, capillary leakage, reduced plasma protein and protein binding. The degree of these alterations may vary considerably and influence the pharmacokinetics (PK) and pharmacodynamics (PD) of antibiotics (Figure 1),^2^, ^3^ potentially leading to suboptimal PK/PD target attainment in up to 60% of the patients.^4^, ^5^ Low antibiotic exposure may be associated with clinical and microbiologic failure, while excessive antibiotic exposure increases the risk of toxicity.^6^, ^7^ To improve target attainment, therapeutic drug monitoring (TDM), which involves the determination of plasma concentrations of a drug as the basis for dosage adjustment, and model‐informed precision dosing (MIPD), which uses population PK models to predict drug exposure and tailor dosing, may play a key role in future dosing strategies.^6^

**FIGURE 2:**
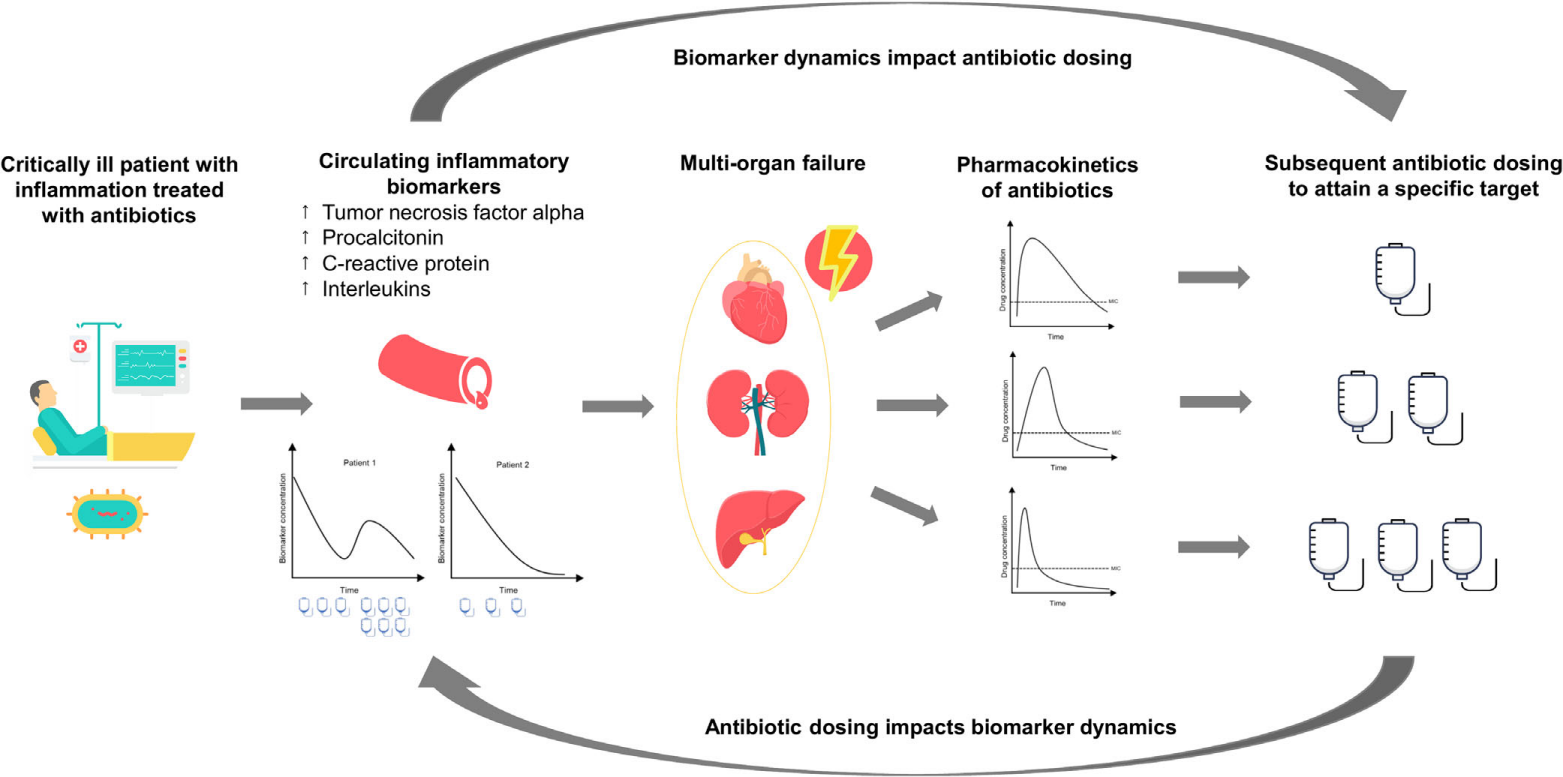
Associations between inflammation and the pharmacokinetics and pharmacodynamics of antibiotics. Example of antibiotics with a time‐dependent mode of action.

Inflammation is a hallmark of critically ill patients and can be assessed through various inflammatory biomarkers. Although there is some evidence suggesting an association of inflammation with antibiotic exposure and clearance,^8^, ^9^ the extent to which the magnitude of the inflammatory response influences the PK/PD variability of antibiotics remains largely unknown. Inflammatory biomarkers such as C‐reactive protein (CRP) and procalcitonin (PCT) have been introduced to measure the degree of inflammation and to assist in determining the initiation and duration of antibiotic treatment,^10^, ^11^, ^12^ but their impact on dose selection is unclear. Integrating inflammatory indicators into drug dosing and monitoring models, thus considering the association between inflammation and PK/PD of antibiotics, could represent the next step towards individualizing pharmacotherapy (Figure 2).^6^, ^13^, ^14^

**FIGURE 3:**
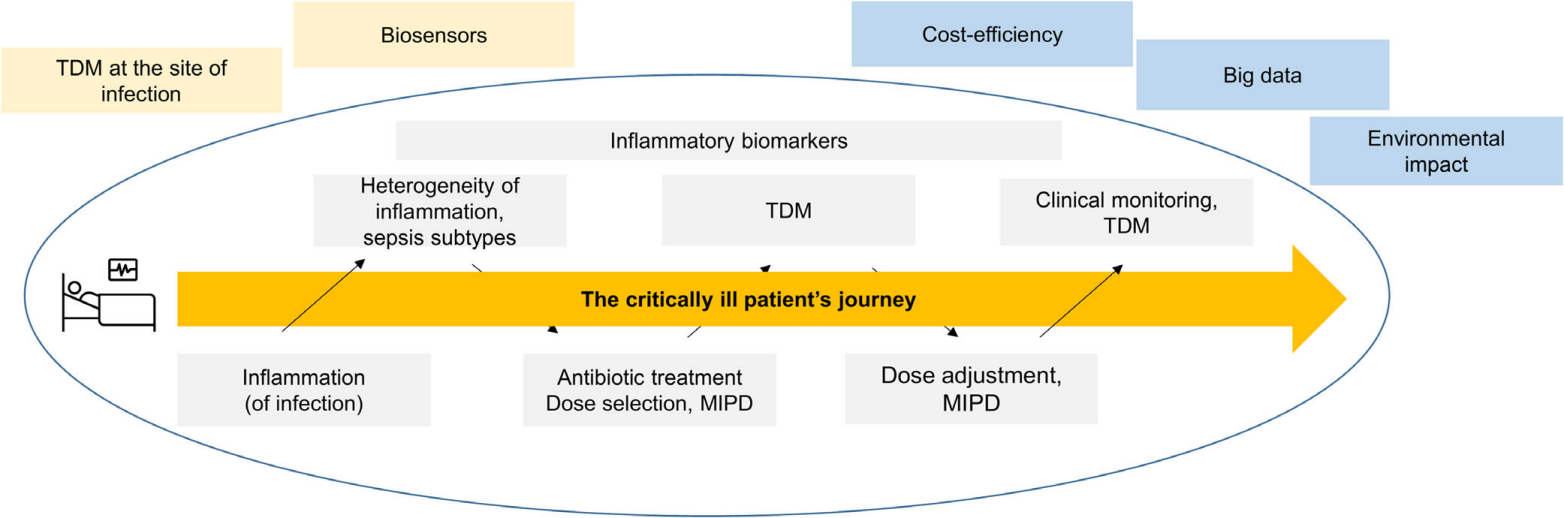
Simplified patients' journey including an overview of the topics addressed in this review, including aspects beyond direct patient care (blue), and additional areas of current research (yellow). MIPD, model‐informed precision dosing, TDM, therapeutic drug monitoring.

This narrative review aims to address the challenges of antibiotic dosing in critically ill patients with inflammation. It seeks to raise awareness of these difficulties and to highlight key open questions that should be the focus of future research. Through the lens of a patient's journey, we guide the reader from the concepts of inflammation, its subtypes and biomarkers to antibiotic dosing, PK/PD of antibiotics, TDM and MIPD, along with the current evidence supporting its use in clinical practice (Figure 3). Finally, we address current and future challenges, including the use of big data to identify patients potentially benefitting from TDM, the cost‐effectiveness of TDM, the importance of environmental sustainability aspects when implementing TDM programmes in the clinics, and novel approaches to perform TDM.

## 2 INFLAMMATION IN CRITICALLY ILL PATIENTS

In critically ill patients, including those with sepsis, inflammation emerges as a defining feature. For decades, dysregulation of the innate immune response, resulting in elevated concentrations of proinflammatory mediators in plasma, has been implicated as a driving force behind the development of multiorgan failure (Figure 1).^15^, ^16^ However, in recent years, several sources of heterogeneity regarding the inflammatory response to sepsis have been identified.^17^ First, there exists considerable variation in the plasma concentration of proinflammatory mediators such as tumour necrosis factor‐α and interleukins, and acute‐phase reactant proteins such as CRP and PCT which are typically recognized as important indicators of hyperinflammation. Second, a one‐dimensional representation of the inflammatory system insufficiently describes the complex interactions between various cell types. Cellular analyses and functional testing have revealed that patients may show signs of hyperinflammation, immunosuppression, or even both simultaneously.^18^ Further complexity is added by our inability to assess whether such changes are appropriate or dysregulated. The immunological response is also highly compartmentalized with, for example, discordant profiles of inflammatory markers in the alveolar space and the systemic circulation, even in cases of disrupted lung barrier function.^19^ Furthermore, time‐related changes in inflammatory patterns remain understudied and contribute to the observed heterogeneity and limited reproducibility in trials. A comprehensive characterization of these heterogeneous phenotypes (e.g., of sepsis), and a better knowledge of the regulation and interplay between pro‐ and anti‐inflammatory mediators is imperative for better understanding the inflammatory profile of patients and its implications on treatment success and outcome.

The most compelling evidence to unravel the biological heterogeneity of inflammatory responses has emerged from biological phenotyping efforts, which involve unbiased analyses of candidate biomarkers or high‐throughput biological data, sometimes in combination with clinical data. Cluster analyses of transcriptomic data from peripheral blood leucocytes of critically ill adults with sepsis have identified 2 sepsis response signatures (SRS1 and SRS2).^20^, ^21^ The SRS1 subtype is characterized by immune exhaustion and increased 14‐day mortality, whereas SRS2 is associated with a relatively immunocompetent status. An unsupervised cluster analysis of transcriptomic data from multiple other sepsis studies has identified 3 distinct subtypes with divergent clinical outcomes, referred to as the *inflammopathic*, *adaptive* and *coagulopathic* subtypes.^22^ These findings suggest that the future of personalized medicine in critically ill patients may revolve around the identification and treatment of biologically distinguishable treatable traits of sepsis. Moreover, they underscore the substantial heterogeneity present in inflammation.

## 3 PK/PD OF ANTIBIOTICS AND TDM IN CRITICALLY ILL PATIENTS

In routine practice, clinicians commonly base dosing decisions on a select number of individual patient factors such as weight and/or end organ function. These limited, routinely available attributes such as creatinine clearance, are commonly implemented into easy‐to‐use dosing nomograms, although the reliability of markers such as creatinine clearance in critically ill patients has been questioned.^23^ The significant variability in exposure and response observed in critically ill patients as a function of PK and PD changes (Figure 1) warrants a more individualized approach to dosing to achieve therapeutic success and limit toxicity.^24^, ^25^, ^26^

**FIGURE 4:**
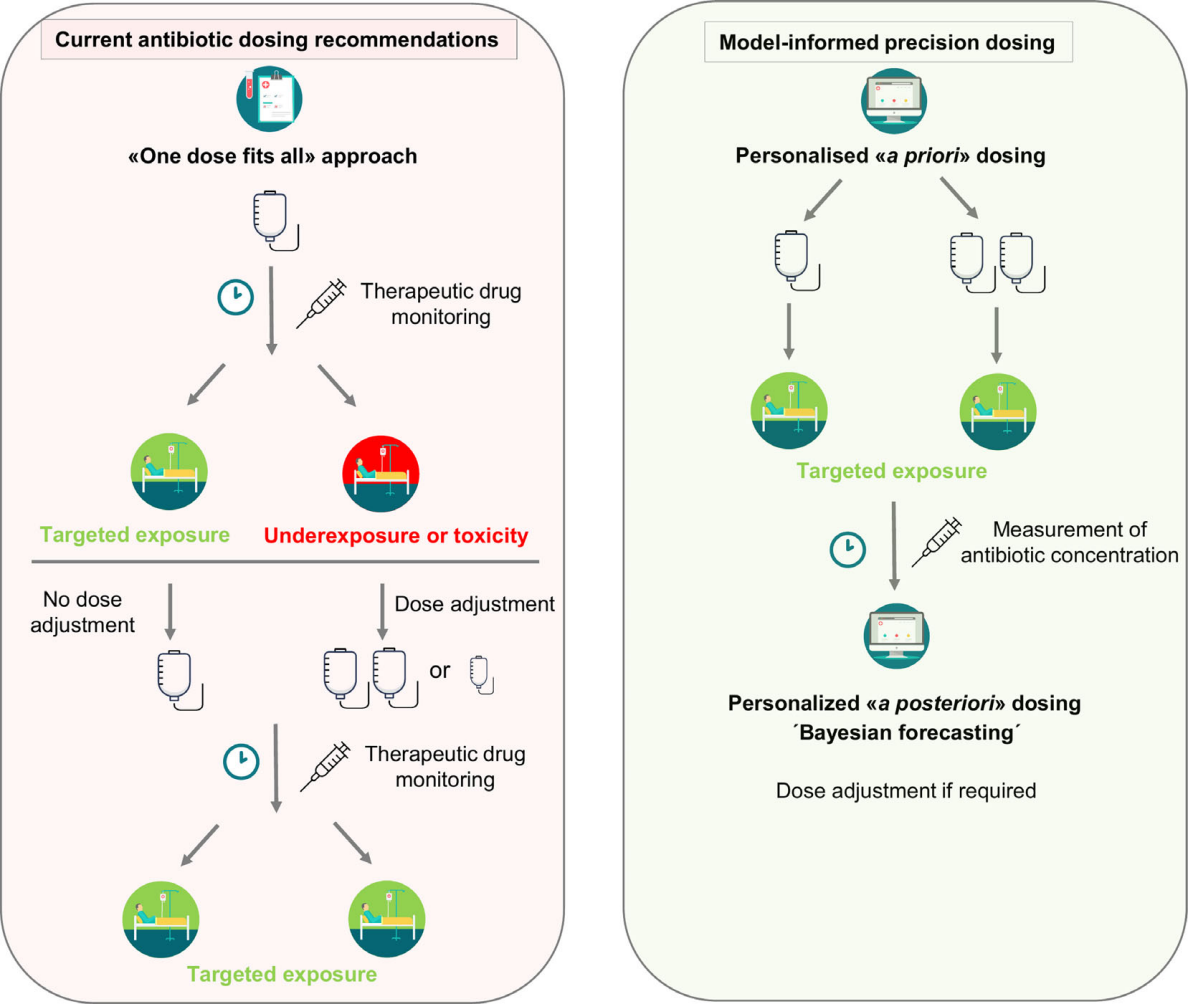
Standard antibiotic dosing recommendations (left) and dosing according to model‐informed precision dosing (right). A priori dosing is based on population pharmacokinetic models, patient characteristics and dosing history and aims to optimize target attainment before initiation of antibiotic treatment. A posteriori dosing additionally considers a patient's individual measured plasma drug concentration(s).

TDM is one strategy for antibiotic dosing individualisation. It involves antibiotic concentration measurement to determine an individual dose associated with favourable clinical outcome. Several limitations of traditional TDM exist.^6^ Sampling typically occurs at a steady state 1–3 days into therapy (e.g. for glycopeptides and β‐lactam antibiotics), which is outside the *golden hours* of sepsis. The exact timing of TDM samples (e.g., minimum plasma concentration) is crucial, but difficult to achieve in clinical practice. If the complete PK profile of a patient is to be characterized, multiple blood draws are required. TDM is most well‐equipped to handle dose–exposure linearity. Dose adjustment for an antibiotic with nonlinear PK is difficult with traditional dosing nomograms and TDM. Overall, while more individualized than standard approaches, TDM remains a reactive response to an existing dosing regimen rather than a proactive method such as MIPD, which aims to optimize an individual dose before or during therapy (Figure 4).

## 4 INDIVIDUALIZING ANTIBIOTIC DOSAGE THROUGH MIPD

MIPD uses population‐driven mathematical models to predict individualized antibiotic dosing.^6^ Population PK or PK/PD models describe and predict the distribution of PK parameters in a set of data observed in a population (e.g. concentration–time profiles), and appraise the individual patient's PK and PD profile.^27^ Empirically driven by the data, parameters are estimated both for the population and the individual. Importantly, the variability between patients with respect to their individual parameters and their PK/PD profiles can be quantified and explained by covariates, which are influential patient‐ or study‐specific characteristics (e.g., age, renal function, body weight) that can be considered for dosing individualisation.

Using a population PK(/PD) model and patient characteristics, MIPD allows a priori population predictions and dosing recommendations up front, thereby limiting the delay to optimal therapy in critically ill patients (Figure 4).^6^ The embedded population models may include a higher number and complexity of covariates than would otherwise be available to inform dosing. As soon as drug concentrations become available, individual model parameters and PK(/PD) profiles can be determined (Bayesian forecasting), together with an individual dosing regimen. Even a single drug concentration can be used to guide dosing regimen individualisation. Unlike traditional TDM practices, the sample(s) can be collected at any time point during a dosing interval. Population models can not only be used to predict drug concentrations for an individual patient, but potentially also toxicity or treatment response.^28^ Repeated measurements of biomarkers as surrogates for inflammatory response may also be included in population models (Figure 2).^6^, ^13^ The choice of a model appropriate for the target population is crucial and presents one of the biggest challenges associated with MIPD. Ideally, the patient to receive MIPD is well represented by the patient population underlying the model to receive an appropriate dosing recommendation. In a retrospective analysis, the performance of 24 PK/PD models was investigated in critically ill patients treated with piperacillin/tazobactam. It showed that not all models may perform equally and that factors such as the mode of administration (intermittent bolus *vs*. continuous infusion), sex or renal replacement therapy should be considered when selecting a model.^29^ Models covering a patient population spanning broad characteristics up to generic metamodels combining diverse populations and/or indications might be a promising next step to increase the reliability of PK/PD models.^30^ Real‐world antibiotic concentration data collected as part of MIPD could later be used for a continuous learning approach to these PK (Bayesian) models.^31^

## 5 CURRENT EVIDENCE FOR THE USE OF TDM AND MIPD IN CRITICALLY ILL PATIENTS

Previous evidence regarding antibiotic TDM and MIPD has primarily focused on target attainment, while several prospective studies also found benefits regarding clinical (e.g., therapy duration, hospital length of stay, nephrotoxicity) and pharmacoeconomic outcomes.^6^ In critically ill patients, dose individualisation of vancomycin, various β‐lactams, and ciprofloxacin was associated with a 1.4‐fold improved target attainment and a 24% reduction in treatment failure.^24^ Randomized controlled TDM and MIPD trials are still scarce and warranted.^32^, ^33^ The only two large multicentre randomized‐controlled trials to date failed to demonstrate the superiority of TDM and MIPD of β‐lactams and ciprofloxacin with respect to the primary outcome parameters.^34^, ^35^ A *posthoc* analysis indicated a benefit for certain subpopulations such as patients with a Sequential Organ Failure Assessment Score (SOFA) <8 or those receiving a dose recommendation within 24 h. However, this analysis also showed that MIPD was associated with increased mortality in patients with a SOFA score >8, an increased intensive care unit (ICU) length of stay in patients with a SOFA <8, and those receiving MIPD for ceftriaxone. Although not powered for this secondary analysis, these results emphasize the importance of identifying target subgroups benefitting from TDM and MIPD.^36^

## 6 BIOMARKERS TO GUIDE ANTIBIOTIC TREATMENT

Biomarkers such as CRP, PCT or interleukins are used to evaluate the degree of inflammation and disease severity in patients with sepsis. Furthermore, they are used to identify bacterial infections, monitor the host's response to antibiotic treatment and guide the duration of antibiotic treatment.^12^, ^37^ Biomarkers may also play a significant role in stratifying patients into those with a hyperinflammatory status and those with an immunosuppressive status. This differentiation is informative for early recognition of organ dysfunction and treatment response.^38^, ^39^ Currently, CRP and PCT are the most widely used and studied biomarkers in sepsis.^38^

We hypothesis that inflammation may impact the PK/PD of antibiotics in numerous ways. First, inflammation may serve as a surrogate for overall organ dysfunction. The magnitude of inflammation may be associated with disease severity and the extent of organ dysfunctions, such as those of the kidneys or the liver.^39^ By including inflammatory biomarkers as an additional parameter in PK/PD models, dosing recommendations may become more appropriate due to the indirect representation of dysfunctional organs by the inflammatory biomarker (Figures 1 and 2). Second, inflammatory biomarkers may directly influence the metabolism of drugs.

The impact of inflammation on antimicrobial drug concentration has mainly been studied in the antifungal voriconazole showing that increased CRP plasma concentrations were associated with higher voriconazole concentrations in paediatric^40^ and in adult patients.^41^, ^42^, ^43^, ^44^ Consistently, a CRP concentration of >100 mg/L,^42^, ^44^, ^45^ has been described above which voriconazole metabolism decreases substantially. In a voriconazole PK model including >1000 voriconazole serum concentrations, CRP was identified as a significant covariate demonstrating that with every 150 mg/L increase in CRP, the maximum voriconazole concentration halved.^41^ In patients treated with antibiotics, first biomarker models have been based on in vitro or animal studies focusing on CRP, PCT, interleukin‐6 and tumour necrosis factor‐α.^37^ In vitro, studies investigating the association between inflammation and antibiotic concentrations are scarce (Table 1). Overall, the number of PK models including inflammation biomarker as covariate in the available is very limited. Negative and positive associations between the inflammation biomarker and the parameter investigated have been reported (Table 1). When interpreting these results, it should be considered that e.g., the production of CRP may be impaired in patients with hepatic dysfunction or patients who receive immunosuppressive drugs such as tocilizumab,^55^, ^56^ This should be scrutinized when including CRP as a covariate in PK models.

**TABLE 1:** The impact of inflammation on antibiotic concentrations.

Antibiotic studied	Year	Study design	PK model included	Patient group	Age	Patient population and site of infection	Number of patients	Biomarker	Parameter	Association between biomarker and parameter	Influence on antibiotic concentration
Linezolid^46^	2016	Prospective	Yes	Adult	58 years (IQR 28–84)	Critically ill patients with severe infections	52	Fibrinogen	CL and serum concentrations	Positive (CL) Negative (serum concentrations)	Higher fibrinogen was associated with a decrease of linezolid concentrations
Piperacillin/ Tazobactam^47^	2021	Retrospective	No	Adult	61 ± 16 years	Critically ill, patients treated with piperacillin/tazobactam	160	CRP and IL‐6	Plasma concentration	Positive	High CRP indicated toxic concentrations. Low CRP might indicate subtherapeutic antibiotic concentrations. Low IL‐6 might indicate subtherapeutic antibiotic concentrations
Amikacin^48^	2022	Prospective	Yes	Adult	58.8 ± 12.9 years	Critically ill patients with intra‐abdominal sepsis	21	ESR	Vd	Positive	Higher ESR was associated with higher Vd and clearance of amikacin
Temocillin^49^	2022	Retrospective	Yes	Adult	56 years (IQR 36–80)	healthy individuals, non‐ICU patients with UTI, ICU patients with suspected/confirmed ventriculitis, patients with sepsis/septic shock	47	CRP	Protein binding	Negative	High CRP was associated with higher free unbound temocillin concentration
Meropenem^50^	2022	Retrospective	Yes	Adult	56 years (IQR 45–65)	Critically ill patients with nosocomial ventriculitis	51	IL‐6 in CSF	Serum and CSF concentration	Positive	High IL‐6 in CSF was associated with higher CSF meropenem concentrations
Meropenem^9^	2022	Prospective	Yes	Adult	63 years (IQR 38–76)	Critically ill patients treated with meropenem	12	CRP	CL	Negative	Higher CRP was associated with decreased meropenem clearance
Vancomycin^51^	2022	Prospective and retrospective	No	Neonates	post‐natal age: 12.5 days (IQR 7–23)	Critically ill patients treated with vancomycin	52	CRP	Plasma concentration	Negative	Higher systemic inflammation was associated with lower vancomycin concentration
Temocillin^52^	2024	Prospective	Yes	Adults	Healthy: 27 years (IQR 23–55) UTI: 72 years (IQR 35–91) Ventriculitis: 55 years (IQR 20–60) Sepsis, ICU: 56 years (IQR 21–80)	healthy volunteers, patients with UTI, patients with ventriculitis, patients with sepsis (ICU)	74	CRP	CL Total number of binding spots (B_max_)	Negative	High CRP was associated with lower B_max_ (leading to higher unbound temocillin concentrations). Higher CRP was associated with lower temocillin CL
Vancomycin^53^	2024	Retrospective	Yes	Adults	79 years (IQR 50–86)	Non‐ICU patients treated with vancomycin	124	CRP	Blood concentration	Positive	High CRP was associated with increased vancomycin trough concentrations
β‐lactam antibiotics and ciprofloxacin^54^	2024	Retrospective	No	Adults	64 years (IQR 55–71)	Critically ill patients treated with a β‐lactam or ciprofloxacin	306	PCT	Blood concentration	Positive	High PCT was associated with higher antibiotic concentrations

Abbreviations: CL, clearance; CRP, C‐reactive protein; CSF, cerebral spinal fluid; ESR, erythrocyte sedimentation rate; ICU, intensive care unit; IL, interleukin; IQR, interquartile range; PCT, procalcitonin; PK, pharmacokinetic; UTI, urinary tract infection; Vd, volume of distribution.

Before biomarkers may be used to guide antibiotic dosing, these results should be confirmed, and more models focusing on antibiotic dosing and the dynamics of inflammatory biomarkers should be developed. Nonetheless, these findings underline that biomarkers may play an informative role as covariates in future PK/PD models.

## 7 TDM AT THE SITE OF INFECTION

Currently, blood (plasma/whole blood) is the preferred specimen for determining antibiotic concentrations. Its collection is standard of care, performed regularly and sample processing procedures are well‐established. However, plasma concentrations can only act as a surrogate for drug concentrations at the sites of infection, i.e., tissue and body fluids such as peritoneal fluid or cerebrospinal fluid (CSF). This poses a particular challenge for infections at sites with limited antibiotic penetration. For example, in patients with ventriculitis or meningitis, β‐lactams and vancomycin exhibit variable penetration into the CSF. Therefore, determining drug concentrations in both plasma and CSF may be needed to develop PK/PD models able to predict antibiotic CSF concentrations and optimize antibiotic treatment.^57^

For pulmonary infections, the determination of antibiotic concentrations in the epithelial lining fluid (ELF) has been proposed as a matrix to determine antibiotic activity.^58^ However, ELF/plasma concentration ratios can vary considerably between different antibiotics based on the physicochemical characteristics of the drug. Furthermore, the availability of ELF is limited due to the invasive collection technique (bronchoalveolar lavage) required, and the necessary experience of the investigators.^58^ Microdialysis and certain biosensors, or a combination of both, may be used to measure drug concentrations in the skin or other tissues, potentially offering more precise information about antibiotic concentrations at the site of infection.^59^, ^60^ These data could inform the development of more complex and mechanistic models, including physiology‐based PK models or quantitative systems pharmacology models, to predict antibiotic exposures at the site of infection, and to unveil mechanisms of toxicity. They could also be analysed using hybrid machine learning/PK approaches and artificial intelligence algorithms to guide antibiotic dosing more accurately. However, these approaches have yet to be validated, and plasma/tissue ratios may vary widely and be influenced by pathophysiological changes and inflammation.

## 8 BIG DATA INCLUDING PK/PD AND INFLAMMATORY DATA TO OPTIMIZE DOSING

The implementation of PK/PD‐based dosing strategies in intensive care medicine poses significant challenges due to the heterogeneity of sepsis definitions and markedly altered PK compared to patients in general wards. At the same time, intensive care medicine is at the forefront of harnessing substantial amounts of diverse, routinely collected, and device‐generated healthcare data. These data include treatment information and information on parameters frequently altered in critically ill patients, such as laboratory measurements, amount of volume resuscitation, haemodynamic monitoring and organ support data.^61^ While these big data bear the potential to improve PK/PD‐based antibiotic dosing in critically ill patients, several important hurdles need to be overcome to effectively employ these data.

First, these big data may facilitate the identification of patients, patient phenotypes or disease phenotypes that may benefit the most from PK/PD‐based antibiotic dosing. They can also help to identify (digital) biomarkers and improve patient inclusion in clinical trials. Clustering techniques, for example, have found subgroups of septic patients that correlated with host–response patterns and even clinical outcomes.^62^ In addition, novel methodologies such as machine learning are especially apt to discover new patterns in large volumes of diverse data^63^ or improve the availability of pharmacological parameters that may not be directly measured.^64^

Notably, there is an increasing interest in target trial emulation to simulate randomized clinical trials on observational data. While these methods are still in their infancy, they may bridge the gap to individualized PK/PD therapies, either in addition to or in conjunction with randomized‐controlled trials.^65^ For big data strategies to thrive in the domain of PK/PD in the ICU, certain prerequisites should be met. The necessary data need to be available and of sufficient quality, accessible in near real‐time and extensively scrutinized for potential biases. Solutions derived from these analyses must then be implemented into the clinical workflow. Although electronic health records present a wealth of health data, many elements, such as diagnoses, are frequently only recorded in unstructured formats such as clinical notes. While large language models are gaining much attention as tools to extract these data, clinical validation remains a challenge in evaluating their true potential. In addition, these methodologies are trained on historical data and may perpetuate human biases that are inevitably and often inadvertently recorded in these data. It is therefore crucial to capture factors that drive this data inequity to continuously evaluate these data and model results. Lastly, implementation science and involvement of the medical community are paramount for realizing clinically impactful technology at the bedside and harnessing the potential of big data PK/PD strategies.

## 9 ENVIRONMENTAL IMPACT AND COSTS OF TDM, AND NOVEL APPROACHES

The primary goal of TDM and MIPD is to optimize antibiotic exposure and to decrease mortality. If this aim can be achieved, the next question may be how to do this in a more sustainable way. The healthcare sector is responsible for 4.4% of the ecological footprint.^66^ TDM requires the use of plastic containers, needles, gloves and chemical reagents such as methanol and acetonitrile, all of which have a substantial impact on the environment.^67^ To make TDM more sustainable, measures such as collecting empty chemical packaging for refilling purposes and repurposing of leftover samples for TDM could be implemented.^68^, ^69^ Although these efforts may have a minuscule impact individually, they show the importance of reconsidering established concepts and replacing routine processes with more sustainable alternatives.

When evaluating the overall cost and health benefits, TDM can decrease health expenditures by reducing drug toxicity rates and hospital length of stay.^70^ Streetman *et al*. demonstrated a 60% decrease in aminoglycoside‐related renal injury when TDM was performed, resulting in savings of up to USD 100 000 for every 100 patients.^71^ Similarly, preventing vancomycin‐related renal injury using two serum samples in PK analyses or MIPD have demonstrated important cost benefits, with savings varying from USD 800–2000 per patient.^72^ A cost–benefit analysis study performed in a resource‐limited setting indicated that vancomycin or aminoglycoside TDM could lead to annual savings of up to USD 125 000–350 000 for every 300 patients.^73^ Additionally, adverse outcomes can result in additional expenditures due to the increased demand for medical support (e.g., ICU admissions). The use of MIPD tools to perform Bayesian forecasting can reduce the number of samples required for processing. Additionally, microsampling methods could be used to collect and transport samples to tertiary care centres where TDM is performed.

However, the ideal approach to TDM of antibiotics in the ICU setting is minimally invasive, provides results immediately (i.e., in real‐time), and allows for easy interpretation at the bedside. Current TDM methods, such as immunoassays, are not suitable for performing continuous, real‐time TDM. In contrast, biosensors, including molecular measurement technologies that employ biorecognition elements to recognize their targets, may be used for point‐of‐care testing and continuous measurements in situ in the body.^74^ Biorecognition elements employed in biosensors include naturally occurring biomolecules (e.g., antibodies, enzymes) and artificially generated biomolecules (e.g., aptamers [short oligonucleotides consisting of RNA or DNA]). Although not yet available, biosensors are a promising and innovative approach to improve patient care.

## 10 CONCLUSION

Antibiotics are a cornerstone of treatment in critically ill patients. Standard recommendations for antibiotic dosing mainly focus on renal function, potentially overlooking other organ dysfunctions, which may impact antibiotic exposure. Given the extensive pathophysiological changes observed in critically ill patients, this approach may be inappropriate and potentially lead to underexposure or toxicity.

Inflammation is frequently observed in critically ill patients, highly variable between patients and over time, thereby impacting the PK and PD of antibiotics. These variabilities and dynamic changes pose challenges in selecting the optimal dosage for a patient. Therefore, TDM should be considered in critically ill patients with inflammation and multiple organ failure to mitigate the risk of inappropriate drug exposure. Whether the routine use of MIPD can improve clinical outcomes in critically ill patients remains a subject for future research. The inclusion of inflammatory biomarkers, as indicators of multiorgan failure, in PK/PD models, and the use of big data capable of predicting antibiotic exposure in critically ill patients, may represent novel approaches to improve antibiotic dosing. Overall, further research is needed to enhance the understanding of the interplay between inflammation and antibiotic PK/PD, with the overall aim of improving antibiotic dosing and patient care.

## AUTHOR CONTRIBUTIONS

Conceptualization: H.E., B.C.P.K., S.D. Methodology: H.E., B.C.P.K., S.D. Investigation: all authors. Resources: all authors. Writing—original draft: all authors. Writing—review and editing: all authors. Visualization: S.D., H.E., B.C.P.K., I.K.M. Supervision: H.E., B.C.P.K. Project administration: S.D., H.E., B.C.P.K. Funding acquisition: B.C.P.K., S.D., E.F.B.

## CONFLICT OF INTEREST STATEMENT

Prof. Birgit C.P. Koch reports grants or contracts unrelated to this work from IMI, European Union, and Prinses Beatrix Foundation; Foundation Coolsingel; a role as unpaid Board member for IATDMCT, ESCMID EPASG, SWAB.

Dr Henrik Endeman reports grants of ZonMw, unrelated to this work, unrestricted grants from Roche, Fisher and Payckel and Ventinova, all unrelated to this work and travel grants and speaker's fees from GETINGE (unrelated to this work).

Dr Erin Barreto reports a consulting relationship with Wolters‐Kluwer (unrelated).

Dr Lucas Fleuren is the cofounder of Medscio.

Dr Lieuwe Bos reports grants from the Dutch lung foundation (Young investigator grant & Dirkje Postma Award), grants from Dutch lung foundation and Health Holland (Public‐Private Partnership grant), grants from IMI COVID19 initiative, grants from Amsterdam UMC fellowship, grants from ZonMW (COVID‐19 Urgency grants and VIDI), ERS Gold Metal for ARDS, and advisory fees paid to the institution from Scailyte, Sobi, Exvastat, Santhera, Pfizer, Astra Zeneca, Janssen en Janssen, all outside the submitted work.

Dr Sophie Stocker reports consultancy arrangements with Nutromic Pty Ltd, 23strands Pty Ltd and Bellberry Pty Ltd which are unrelated to this work.

All other authors declare that they have no conflicts of interest.

## Data Availability

N/A
